# Self-harm among post-natal mothers in Northwest Ethiopia: Implication for policy and practice

**DOI:** 10.3389/fpubh.2022.916896

**Published:** 2022-11-08

**Authors:** Agumas Eskezia Tiguh, Kindu Yinges Wondie, Dereje Nibret Gessesse, Nuhamin Tesfa Tsega, Mastewal Belayneh Aklil, Wubedle Zelalem Temesgan, Marta Yimam Abegaz, Tazeb Alemu Anteneh, Nebiyu Solomon Tibebu, Haymanot Nigatu Alemu, Tsion Tadesse Haile, Asmra Tesfahun Seyoum, Tiruye Tilahun Mesele, Ayenew Engida Yismaw, Goshu Nenko, Birhan Tsegaw Taye, Muhabaw Shumye Mihret, Azmeraw Ambachew Kebede

**Affiliations:** ^1^Department of Clinical Midwifery, School of Midwifery, College of Medicine and Health Sciences, University of Gondar, Gondar, Ethiopia; ^2^Department of Women's and Family Health, School of Midwifery, College of Medicine and Health Sciences, University of Gondar, Gondar, Ethiopia; ^3^Department of Psychiatry, College of Medicine and Health Sciences, University of Gondar, Gondar, Ethiopia; ^4^School of Nursing and Midwifery, Asrat Woldeyes Health Science Campus, Debre Berhan University, Debre Berhan, Ethiopia

**Keywords:** self-harm, post-natal, Northwest Ethiopia, Gondar, associated factors

## Abstract

**Introduction:**

Self-harm is a global public health concern affecting thousands of women. However, it is an under-reported and neglected aspect of maternal health, particularly in developing countries. In Ethiopia, there is a paucity of evidence regarding self-harm, and it is rarely given attention. Therefore, this study aimed to assess the proportion of self-harm and associated factors among postnatal mothers in Gondar city, Northwest Ethiopia.

**Method:**

A community-based cross-sectional study was conducted from 1 July, 2021, to 30 August, 2021, in Gondar city. A cluster sampling technique was conducted to select 858 women who gave birth in the last 12 months. The data were collected using a structured questionnaire through face-to-face interviews. The data were entered into EpiData version 4.6 and exported to SPSS 25 for analysis. The multivariable logistic regression analysis was fitted to identify factors associated with the outcome variable. The level of significant association was determined at a *p*-value of ≤ 0.05.

**Result:**

The proportion of postnatal self-harm was found to be 8.5% (95% CI: 6.7,10.5). Having lower family income (AOR: 2.41, 95% CI: 1.05,5.56), having unplanned pregnancy (AOR: 2.70, 95% CI: 1.53,4.79), experiencing adverse birth outcomes (AOR: 3.11, 95% CI: 1.10,8.83), birth not attended by health provider (AOR: 4.15, 95% CI: 1.76,9.79), experiencing intimate partner violence (AOR: 1.93, 95% CI: 1.12,3.32), and poor decision-making power (AOR: 1.70, 95% CI: 1.02, 2.84) were the variables significantly associated with self-harm.

**Conclusion:**

This study revealed that the proportion of self-harm among postnatal mothers was prevalent. Factors like monthly income of a family, planned pregnancy, birth outcome, birth assistant, intimate partner violence, and decision-making power show an association with maternal self-harm. Antenatal and postnatal self-harm screening as part of the continuum of maternal healthcare is important. Self-harm is also a danger for women who have experienced intimate partner violence or have low socioeconomic economic status, all of which require exceptional mental health assessment.

## Introduction

Self-harm is an act in which a person intentionally initiates a non-habitual behavior that will result in self-harm without the intervention of others, or deliberately ingests a substance over the prescribed or generally recognized therapeutic dosage. The act has the goal of achieving the changes that the subject desires through the actual or expected physical consequences (1). Self-harm ideation is a term used to describe thoughts about intentionally harming oneself, with or without the intent of dying (2), whilst suicidal ideation refers to specific thoughts and images of, as well as preparations for ending one's life. Both can have serious implications for women and children (3).

A woman's life undergoes significant physiological, social, and emotional changes throughout pregnancy and childbirth (4, 5). Mental disorders are frequently typical throughout pregnancy and the postpartum period (6), but most of the time, these are normal. These disturbances could, however, worsen and result in self-harm (7). Pregnant women are among the most vulnerable groups in the community to mental health problems during the COVID-19 pandemic (8). Despite the popular perception that pregnancy protects against self-harm, a new study found that pregnant women are more likely to engage in it (9). There is growing evidence that women in the perinatal period (pregnancy and postpartum) are more prone to self-harm than women in the general population (9). Although direct obstetric causes account for the majority of maternal deaths, indirect factors account for 27.5% of maternal deaths and are on the rise (10). Ending preventable causes of maternal mortality requires the consideration of all reproductive and maternal morbidities and related disabilities (11, 12).

Self-harm is estimated to occur in 75.5% of low- and middle-income countries (13). The mother's morbidity and maternal mental disorders may be linked to obstetric complications and developmental problems in the child (14, 15). Self-harm increases the risk of low birth weight (16) and has less positive effects among infants (17). The majority of maternal deaths due to self-harm occur in the late postpartum period (18).

Improving maternal and newborn health is one of the government's concerns both nationally and globally, which comprises the third component of the SDG (19). Improving the optimal health of women and newborns, as well as reducing maternal and neonatal mortality to as low a level as possible by assessing maternal mental health problems and associated factors, will undoubtedly play a significant role in the achievement of SDG (20).

Although there are few articles regarding maternal mental health problems (depression and a few articles on anxiety) in Ethiopia, there is a lack of evidence on intentional self-harm (21), and there is no routine screening platform for self-harm at maternal healthcare service setups. As a result, this study will fill in the gaps by assessing self-harm among postnatal mothers and identifying individual level factors. Furthermore, this research may provide fundamental evidence for any interventions aimed at improving the mental health of pregnant and postnatal women in the community and developing effective strategies to address preventable maternal mental health mortality and morbidity.

## Methods

### Study design, period, and area

A community-based cross-sectional study was undertaken in Gondar City from 1 July, 2021, to 30 August, 2021. The city is located in the Central Gondar Zone of the Amhara National Regional State. It lies 750 kilometers northwest of Ethiopia's capital, Addis Ababa, and 170 kilometers north of Bahir Dar, the capital of the Amhara National Regional State. According to Ethiopian demographic projections, the city's total population is predicted to be 432,191, of whom 224,508 are women. Approximately 133,477 (30.88%) of the women are of reproductive age. In the city, there is one governmental referral hospital, 8 governmental health facilities, 22 health posts, 1 private primary hospital, and 1 general hospital.

### Study population and eligibility criteria

Permanent resident women who gave birth in the past 12 months and were available during the time of data collection were included. Women who were critically ill and unable to respond throughout the data collection period were excluded.

### Sample size determination and sampling procedure

The sample size was determined using a single population formula considering the following assumptions: the proportion of self-harm 50%, level of confidence 95%, and margin of error 5%. Therefore, the sample size is as follows:

(n) $=\frac{{\left(Z\alpha /2\right)}^{2}\;p(1-p)}{d^{2}}\frac{=\;{\left(1.96\right)}^{2^{*}}0.5(1-0.5)}{(0.05)2}=$ 384. After considering a design effect of 2 and a 10% non-response rate, the final sample size becomes 845.

Seven kebeles were selected by lottery method from the 22 kebeles of Gondar city, a home-to-home visit was conducted in the selected kebeles (clusters). In the selected clusters, all eligible women were interviewed. Finally, because of the nature of cluster sampling, 872 women were interviewed, but we only got complete data from 858 of them.

### Operational definition

#### Intentional self-harm

This is defined as a suicide attempt or self-injurious behavior including cutting, burning, hitting, hanging, overdosing, poisoning, and electrocuting. The study participants were asked whether they have had an intentional attempt to harm themselves during the postpartum period (22).

#### Household decision-making power

The ability of women to act independently on household activities including their own health, children's health, freedom of movement, and control over finance without asking permission from another person. Based on the summative score of variables designed to assess household decision-making power, women who will answer above the mean value will be considered as having higher decision-making power (23).

#### Husband/partner involvement in MNCH services

Based on the summative score of variables designed to assess husband involvement, a score above the mean is considered as involved (24).

#### Intimate partner violence

Intimate partner is considered as the current spouse, cohabited, current boyfriend, former partner, or spouse. If the respondent answers “Yes” to any one of the ranges of sexual, psychological, and physical or any combination of the three coercive acts regardless of the legal status of the relationship with the current/former intimate partner, it will be considered as intimate partner violence (25).

#### Social support

The Oslo Social Support Scale (OSS-3) scores ranged from 3 to 14 with a score of 3–8, poor support; 9–11, moderate support; and 12–14, strong support (26).

#### Depression

Women who were interviewed and scored 10 and above by using Patient Health Questionnaire-9 (PHQ-9) were considered depressed (27).

#### Loneliness

To measure loneliness, the University of California Los Angeles Loneliness Scale (UCLA-3 scale) was used based on the total score obtained. Women who score >28 had loneliness and scored < 28 did not have loneliness (28).

### Data collection procedures and data quality control

The data collection tool was developed by reviewing literature and guidelines (22, 29, 30). Initially, the questionnaire was prepared in English and translated into Amharic for clarity and back to English to ensure its consistency. The data were collected using a structured questionnaire through face-to-face interviews. The questionnaire was assessed by a group of researchers to check for content validity. The questionnaire contains sociodemographic characteristics, obstetric and maternal health service-related characteristics, decision-making autonomy, and intimate partner violence-related questions, and items assessing self-harm, social support, depression, and maternal loneliness. After being trained in the interview technique, seven B.Sc and two M.Sc midwives collected and supervised the data collection, respectively. A pretest was done on 5% of the sample size outside of the study setting to check the response, language clarity, and appropriateness of the questionnaire. During data collection, the questionnaire was checked for completeness daily by the supervisors.

### Data processing and analysis

The data were checked, coded, and entered into EpisData version 4.6 and exported to SPSS^®^ version 25 for further cleaning and analysis. Descriptive statistics are done using tables and words. Binary logistic regression was fitted to identify independent predictors and variables having a *p*-value of < 0.2 were included in the multivariable logistic regression for controlling confounders. In multivariable logistic regression, a *p*-value of ≤ 0.05 with 95% CI for the odds ratio was used to determine the significant association.

### Ethical considerations

Ethical clearance was obtained from the Institutional Review Board (IRB) of the University of Gondar (Reference Number: V/P/RCS/05/2710/2021). A formal support letter was obtained from the administrative offices of each selected kebele. Written informed consent was taken from each study participant after a clear explanation of the purpose of the study.

## Results

### Sociodemographic characteristics

A total of 858 study participants were included in this study, giving a response rate of 98.39%. Two-fifths (40.8%) of the study's participants were between the age of 26 years and 30 years old, with a mean age of 29.53 ± 4.7. More than two-fifths (43.2%) of participants had a college or above educational level. Regarding their occupation, 44.4% were housewives. Most (90.8%) of the respondents were married. More than four-fifths (82.3%) of the study participants were orthodox religious followers. Nearly half (49.3%) of the participants have 5,000 ETB or more in average monthly income. More than half (53.8%) of mothers live with four to five family members (Table 1).

**Table 1:** Sociodemographic characteristics of study participants in Gondar city, Northwest Ethiopia, 2021 (*n* = 858).

**Variable**	**Frequency**	**Percent**
**Age**
≤ 25	183	21.3
26–30	350	40.8
≥31	325	37.9
**Educational status**
No formal education	105	12.2
Primary education	140	16.3
Secondary education	242	28.2
College and above	371	43.2
**Occupation**
Housewife	381	44.4
Merchant	105	12.2
Self employed	99	11.5
Daily laborer	34	4.0
Government employee	239	27.9
**Current marital status**
Married	779	90.8
Unmarried	79	9.2
**Religion**
Orthodox Christian	706	82.3
Muslim	107	12.5
Others*	45	5.2
**Family size**
< 3	243	28.3
4-5	462	53.8
≥6	153	17.8
**Average family monthly income in ETB**
< 1,500	63	7.3
1,501–5,000	372	43.4
≥5,001	423	49.3

^*^Protestant, catholic, Adventist, Jewish.

### Reproductive, obstetrics, and medical-related characteristics of the study participants

In this study, about 57.6% of the women were multiparous. In the most recent pregnancy, 745 (86.8%) of women had planned a pregnancy. More than four-fifths (83.8%) of women received three or more ANC visits, and 68.8% gave birth in a government hospital. Most (96.5%) of the study participants' births were assisted by health professionals. Most (97.4%) of them were live births. Regarding medical status, about 10, 2.2, and 9.2% of the study participants had known medical illnesses, psychiatric problems, or a family history of mental illness, respectively. According to this study finding, 65% of women's husbands or partners are involved in MNCH care and 25.5% have strong social support. Slightly more than two-thirds of women have good decision-making power in household activities, whereas nearly half (48.6%) of women experienced intimate partner violence in the most recent pregnancy and childbirth period. About two-fifths (40%) of the participants develop loneliness, and 11.2% of them develop depression during their recent pregnancy and the postpartum period (Table 2).

**Table 2:** Reproductive, obstetrics, and medical-related characteristics of study participants in Gondar town, Northwest Ethiopia, 2021 (*n* = 858).

**Variable**	**Frequency**	**Present**
**Parity**
Primi Para	364	42.4
Melty para	494	57.6
**Number of ANC visits**
0	23	2.7
1–2	202	23.5
≥3	633	73.8
**Pregnancy was planed**
Yes	745	86.8
No	113	13.2
**Pregnancy was supported**
Yes	794	92.5
No	64	7.5
**Place of delivery**
Government hospital	590	68.8
Health center	210	24.5
Private hospital/clinic	26	3
At home	32	3.7
**Birth attendant**
Health professional	828	96.5
Other than health professional	30	3.5
**Pregnancy outcome**
Live birth	836	97.4
Not live birth, neonatal death, and congenital anomaly	22	2.6
**Number of PNC visits**
0	408	47.6
1	214	24.9
≥2	236	27.5
**Husband/partner involved in MNCH**
No	296	34.5
Yes	562	65.5
**Social support**
Poor	247	28.8
Moderate	392	45.7
Strong	219	25.5
**Women**'**s decision-making power**
Poor	278	32.4
Good	580	67.6
**Intimate partner violence**
No	441	51.4
Yes	417	48.6
**Known medical disorder**
Yes	86	10.0
No	772	90.0
**Known psychiatric problem**
Yes	19	2.2
No	839	97.8
**Family history of mental illness**
Yes	79	9.2
No	779	90.8
**Depression**
No	762	88.8
Yes	96	11.2
**Loneliness**
No	507	59.1
Yes	351	40.9

### Self-harm among postnatal mothers and associated factors

The proportion of self-harm among postnatal mothers was found to be 8.5% (95% CI: 6.7, 10.5).

In the multivariable logistic regression, average family monthly income, pregnancy status, birth outcome, birth attendant, intimate partner violence, and decision-making have shown an association with self-harm.

Women with an average monthly income of < 1,500 ETB were 2.41 times more likely to attempt self-harm than those with an income of more than 5,000 ETB (AOR: 2.41, 95% CI: 1.05, 5.56). The odds of having self-harm were 2.7 times higher among women whose pregnancy was unplanned as compared with their counterparts (AOR: 2.70, 95% CI: 1.53,4.79). Experiencing adverse birth outcomes was found to increase the odds of self-harm by 3.11 times (AOR: 3.11, 95% CI: 1.10, 8.83). Women whose birth was not attended by healthcare providers were 4.15 times more likely to develop self-harm than those women whose birth was attended by healthcare providers (AOR: 4.15, 95% CI: 1.76, 9.79). The odds of self-harm were 1.93 times higher among women who had experienced intimate partner violence during pregnancy than those women who had never experienced intimate partner violence (AOR: 1.93, 95% CI: 1.93, 1.12, 3.32). Women who had poor decision-making power were 1.70 times more likely to experience self-harm than those women who had good decision-making power in the household (AOR: 1.70, CI: 1.02,2.84) (Table 3).

**Table 3:** Bi-variable and multivariable logistic regression analysis of postnatal self-harm and associated factors in Gondar City, Northwest Ethiopia, 2021, (*n* = 858).

**Variable**	**Self-harm**		**COR (95% CI)**	**AOR (95% CI)**
	**Yes**	**NO**		
**Educational status**
No formal education	21	84	4.63 (2.38, 9.00)	1.68 (0.74, 3.82)
Primary	12	128	1.73 (0.82, 3.68)	0.85 (0.36, 2.02)
Secondary	21	221	1.76 (0.93, 3.35)	1.00 (0.48, 2.06)
Diploma and above	19	352	1	1
**Occupation**
Housewife	38	343	1.93 (1.00, 3.69)	0.87 (0.34, 2.24)
Merchant	9	96	1.63 (0.67, 3.94)	1.15 (0.38, 3.49)
Self-employed	7	92	1.32 (0.51, 3.42)	0.70 (0.24, 2.10)
Daily laborer	6	28	3.72 (1.31, 10.58)	0.93 (0.24, 3.61)
Government employee	13	226	1	1
**Family monthly income**
< 1,500 ETB	12	51	3.31 (1.59, 6.93)	**2.41 (1.05, 5.56)**
1,501–5,000	33	339	1.37 (0.81, 2.32)	1.42 (0.82, 2.45)
≥5,001 ETB	28	395	1	1
**Planned pregnancy**
Yes	49	696	1	**1**
No	24	89	3.83 (2.24, 6.54)	**2.70 (1.53, 4.79)**
**Pregnancy supported by family**
Yes	41	753	1	1
No	10	54	3.49 (1.82, 6.67)	1.19 (0.45, 3.09)
**Pregnancy outcome**
Live birth	66	770	1	**1**
Not live birth	7	15	5.44 (2.14, 13.82)	**3.11 (1.10, 8.83)**
**Birth assistant**
Health providers	63	765	1	**1**
Non than health providers	10	20	6.07 (2.72, 13.53)	**4.15 (1.76, 9.79)**
**Known medical illness**
Yes	12	74	1.89 (0.97, 3.67)	1.58 (0.73, 3.45)
No	61	711	1	1
**Known psychiatric problem**
Yes	5	14	4.05 (1.41, 11.58)	1.99 (0.60, 6.53)
No	68	771	1	1
**Family history of mental illness**
Yes	12	67	2.11 (1.08, 4.11)	1.78 (0.87, 3.63)
No	61	718	1	1
**Intimate partner violence**
No	23	418	1	**1**
Yes	50	367	2.48 (1.48, 4.14)	**1.93 (1.12, 3.32)**
**Loneness**
No	36	471	1	1
Yes	37	314	1.54 (0.95, 2.49)	1.09 (0.56, 2.11)
**Depression**
No	58	704	1	1
Yes	15	81	2.25 (1.22, 4.15)	1.01 (0.48, 2.14)
**Social support**
Poor support	29	218	2.78 (1.32, 5.85)	1.59 (0.71, 3.54)
Moderate support	34	358	1.98 (0.961, 4.10)	1.82 (0.85, 3.86)
Strong support	10	209		1
**Decision making power**
No	36	242	2.18 (1.35, 3.54)	**1.70 (1.02, 2.84)**
Yes	37	543	1	**1**
**Husbanded involvement**
No	38	258	2.22 (1.37, 3.60)	1.38 (0.80, 2.39)
Yes	35	527	1	1

The bold values indicate *p* ≤ 0.05.

## Discussion

This study is aimed at assessing the proportion of self-harm and associated factors among postnatal mothers. Thus, the study showed that 8.5% of postnatal mothers had experienced self-harm in their last pregnancy and childbirth. The finding is consistent with the reports from South Africa (7%) (31), United Kingdom (7.9%) (32), Canada (10.4%) (33), and Japan (9.1%) (34). However, the findings of this study are lower than those of studies conducted in Sri Lanka (27.4%) (7) and the USA (19.3%) (35) and (22.3%) (36). This discrepancy might be due to the difference in sociodemographic characteristics, time gaps, and sample size. For example, the study conducted in the USA incorporated white, African American, and Asian ethnicities, and a majority of the study participants were educated to graduate level or higher. Furthermore, the aforementioned studies were conducted at the health institution level, whereas our study was conducted at the community level. Some of the previous studies were also done on women with identified depression. Mothers with other mental health problems, such as depression and stress are highly prone to self-harm (29, 33, 37, 38).

On the other hand, the prevalence of self-harm in this study is higher than in the study conducted in Australia (4.6%) (39). The inconsistency could be explained by sociodemographic and cultural differences and the data collection procedure differences. The Australian study was mainly focused on women who became pregnant and gave birth for the first time, whereas our study included both primiparous and multiparous women.

The factors, such as low family monthly income, unplanned pregnancy, adverse pregnancy outcomes, intimate partner violence, and poor decision-making power were significantly associated with self-harm among postnatal mothers. Those women having an average monthly income of < 1,500 ETB were 2.4 times more likely to attempt self-harm than those women having a monthly income of >5,000 ETB. This finding is in line with a study conducted in Japan (34). The possible explanation could be due to the fact that lower incomes cannot fulfill vital needs and women with lower incomes might not be able to satisfy their cost of living, which may create additional stress for women that can lead to self-harm. Women who have a better monthly household income have higher autonomy over their health (23).

This study found that self-harm was 2.7 times more common in women having an unplanned pregnancy than those women having a planned pregnancy. This finding is supported by a study conducted in the United Kingdom (40). Unintended pregnancy affects women's mental health (41). The possible reasons could be that most unplanned pregnancies tend to be unwanted which constitutes extra worry that might lead to self-harm. To reduce the number of unplanned pregnancies, it is beneficial to minimize the unmet need for family planning by expanding its coverage to all reproductive-age women, which may reduce women's stress that leads to self-harm. The odds of self-harm were 3.11 times higher among women having an adverse pregnancy outcome than those having a normal birth outcome. This finding is supported by evidence (42). This might be due to the reality that a woman becoming expecting to have a healthy neonate through normal birth, but if they face a sudden bad obstetric outcome (i.e., stillbirth), their mental health status will be affected and lead to self-harm. In addition, some maternal mental health problems are associated with premature delivery, low birth weight, stillbirth, and infant deaths (15). It is essential to increase the quality of obstetric care practices to minimize the negative outcome of pregnancy. In addition, adequate counseling and reassurance are crucial for women with adverse pregnancy outcomes, which will reduce feelings of self-harm.

Women whose birth was attended by someone other than a healthcare professional were 4.5 times more likely to experience self-harm than those assisted by healthcare providers. This can be explained by mothers receiving support and counseling from health providers during their ANC follow-up and childbirth. Thus, expanding maternal health services utilization including institutional delivery by skilled healthcare providers may decrease maternal self-harm.

Another important finding of this study is that women violated by their intimate partners were 1.93 times more likely to practice self-harm than those who have not experienced intimate partner violence. This finding is in line with the study conducted in Australia (39), and studies also showed that IPV is associated with suicidal ideation in perinatal women (31). Evidence suggests that intimate partner violence is a promoter of self-harm (43). This could be explained by women with intimate partner violence may attempt self-harm as a way to express unpleasant emotions brought on by abuse, or as a last option to escape through death when they had exhausted all other alternatives and could no longer tolerate the violence. In order to alter cultural norms and attitudes and empower women about intimate partner violence, it is essential to raise community awareness of the harmful effects of intimate partner violence through education campaigns and the dissemination of information through the media. Furthermore, the healthcare system's curriculum should include IPV screening, especially in antenatal and postnatal care. Since maternity care offers a chance to identify women who experience intimate partner violence and a place that is appropriate for discussing abuse issues. This might decrease mothers' self-harm.

Furthermore, women who had poor decision-making power were 1.7 times more likely to attempt self-harm than those who had good decision-making power. This might occur if the mother does not make decisions about household consumption, the woman may feel disliked and inferior in the family. As a result, the woman may later harm herself. Poor decision-making power has already been linked to perinatal mental health problems (44). Women's empowerment and participation in decision-making will increase the perception of strong social support, which will lessen self-harming sentiments. Assessing women's mental health status at maternal healthcare services is an important technique for determining women who are at an increased risk of postnatal self-harm. Healthcare providers should be encouraged to inquire about all postnatal women's personal mental health histories and could make mental health assessments.

### Implication for policymakers

This study collects data on maternal self-harm in the postpartum period and related factors. The findings of this study are being used to identify pertinent stakeholders and health policymakers who pay close attention to maternal mental health issues, including the use of screening tools throughout the continuum of care for mothers. The significance of this study for public health is that it illuminates the causes of self-harm, allowing for the development of preventative strategies to address the problem and its consequences. The clinical significance of this study is to inform health professionals, health managers, and other policymakers about the risk factors for self-harm among postpartum women so that they can take appropriate action to reduce the risk and increase their efforts to develop appropriate strategies. The findings also urge expanded education and community awareness as a package of community services, strengthening the ability of women to make decisions in the home and fostering social support both inside the home and in the community.

### Limitation

Some limitations need to be acknowledged. Because of the interviewer-administered data collection, there is a risk of social desirability bias, in which women may underreport self-harm and other mental health problems, in part owing to stigmatization. To minimize this a private place was selected and the women were interviewed alone during data collection.

## Conclusion

This study revealed that the proportion of self-harm among postnatal mothers was significant. Factors like family monthly income, planned pregnancy, birth outcome, birth assistant, intimate partner violence, and decision-making power show an association with self-harm among postnatal mothers. Thus, the Federal Ministry of Health, the Regional Health Bureau, health service managers, and health professionals should expand maternal health services with self-harm screening, intervention, and prevention strategies. Community health extension workers better assess maternal mental health status and make referral linkage to healthcare services. Community leaders better to empowering women economically and socially.

## Data availability statement

The raw data supporting the conclusions of this article will be made available by the authors, without undue reservation.

## Ethics statement

The studies involving human participants were reviewed and approved by Institutional Review Board (IRB) of the University of Gondar (Reference Number: V/P/RCS/05/2710/2021). The patients/participants provided their written informed consent to participate in this study.

## Author contributions

AT and KW conceived and designed the experiments, performed the experiments, analyzed and interpreted the data, and wrote the paper. DG, NT, MBA, WT, MYA, TA, NT, HA, TH, AS, TM, AY, MM, GN, BT, and AK performed the experiments, analyzed and interpreted the data, and wrote the paper. All authors contributed to the article and approved the submitted version.

## Conflict of interest

The authors declare that the research was conducted in the absence of any commercial or financial relationships that could be construed as a potential conflict of interest.

## Publisher's note

All claims expressed in this article are solely those of the authors and do not necessarily represent those of their affiliated organizations, or those of the publisher, the editors and the reviewers. Any product that may be evaluated in this article, or claim that may be made by its manufacturer, is not guaranteed or endorsed by the publisher.
